# Association of hypomagnesemia with chronic use of proton-pump inhibitors in patients visiting a tertiary care hospital in Peshawar, Pakistan

**DOI:** 10.12669/pjms.41.5.11993

**Published:** 2025-05

**Authors:** Khushal Nadir Hadi, Niktash Khan Hadi, Ayesha Bangash, Fawad Rahim

**Affiliations:** 1 Khushal Nadir Hadi, Department of Medicine, Hayatabad Medical Complex, Phase 4 Hayatabad, 25200, Peshawar, Pakistan; 2 Niktash Khan Hadi, Shaukat Khanum Memorial Cancer Hospital and Research Centre, Phase 5 Hayatabad, Peshawar, Pakistan; 3 Ayesha Bangash, Department of Medicine, Hayatabad Medical Complex, Phase 4 Hayatabad, 25200, Peshawar, Pakistan; 4 Fawad Rahim, Department of Medicine, Hayatabad Medical Complex, Phase 4 Hayatabad, 25200, Peshawar, Pakistan

**Keywords:** Hypomagnesemia, Magnesium, Pakistan, Proton pump inhibitors

## Abstract

**Objective:**

To determine the association between hypomagnesemia and chronic use of proton pump inhibitors (PPIs) among patients visiting a tertiary care hospital.

**Method:**

This cross-sectional study was conducted in the Department of Medicine at Hayatabad Medical Complex, Peshawar, Pakistan from July 2024 to December 2024. A total of 303 patients aged 18 years and above, who had used a PPI for at least one month, were enrolled. Age, gender, body mass index, patient setting, duration and type of PPI used, and the presence of hypertension and diabetes mellitus were documented. Serum magnesium levels were measured for all participants. The data were analyzed using SPSS.

**Results:**

Hypomagnesemia was observed in 51.5% (n=156) patients. Patients with hypomagnesemia had a significantly higher mean age compared to those without (48.9±12.3 vs 38.2±11.4 years, p <0.001). On multivariate analysis, PPI use for more than six months (AOR: 58.1, 95% CI: 22.2–152.5, p<0.001), diabetes mellitus (AOR: 23.1, 95% CI: 6.4–82.6, p<0.001), and hypertension (AOR: 3.2, 95% CI: 1.4 – 7.1, p<0.001) were identified as independent predictors of hypomagnesemia. No significant differences in serum magnesium levels were found among patients using different types of PPIs (p=0.131).

**Conclusion:**

Hypomagnesemia was found in 51.5% of patients, irrespective of PPI type. Significant risk factors included advanced age, prolonged PPI use, diabetes mellitus, and hypertension. Raising awareness among healthcare professionals about PPI’s potential adverse effects and advocating for prudent prescription practices is essential.

## INTRODUCTION

Magnesium is the body’s fourth-most abundant cation and is responsible for the action of crucial enzymes.^1^ Many patients with magnesium deficiency may remain asymptomatic.^2^ However, there are various clinical manifestations of reduced magnesium levels in the body that may range from decreased appetite, nausea, tiredness, generalized fatigue, muscle cramps, and paresthesia to more serious and, in some cases, fatal complications such as arrhythmias, seizures, coronary artery spasm, and sudden death.^3^

In recent years, various studies have linked proton pump inhibitors (PPIs) to hypomagnesemic hypoparathyroidism and also described them as abettors to diuretic-induced hypomagnesemia.^4^,^5^ It is postulated that PPIs lead to reduced absorption of magnesium in the distal intestine; the mechanism behind this decrease is linked with proteins present on the surface of intestinal cells called the transient receptor potential melastin 6 (TRPM 6) and TRPM 7.^6^ These proteins function more effectively in an acidic environment. However, PPIs, dampen TRPM activity resulting in reduced magnesium absorption and hypomagnesemia.^7^

Studies on hypomagnesemia in PPI users have shown varying results. The prevalence of hypomagnesemia in PPI users varies widely across these studies, with proportions reported between 3.6% and 55.2%.^8^,^9^ In Pakistan, limited data is available, with a case report by Zafar et al. that highlighted hypomagnesemia in a patient with long-term PPI use.^10^ A cross-sectional study by Siddiqi et al. comparing PPI use and serum magnesium levels had insignificant results, reporting hypomagnesemia in their study population at 2.3%.^11^ Some studies did not account for confounding factors or use both outpatient and inpatient data when analyzing hypomagnesemia in PPI users.

There is an inappropriate overuse of over-the-counter PPIs in Pakistan.^12^ Given the widespread use of PPI, the clinical consequences of hypomagnesemia, and the limited evidence from our population, this is the first study aimed at determining the frequency and predictors of hypomagnesemia in patients using PPIs.

## METHODS

This cross-sectional study was conducted in the Department of Medicine at Hayatabad Medical Complex, Peshawar, Pakistan. This study (n=303) was carried out from July 2024 to December 2024. The sample size was calculated by taking the prevalence of hypomagnesemia in patients with chronic PPI use as 27% with 95% confidence levels and a 5% margin of error.^13^

### Inclusion & Exclusion Criteria:

Patients, 18 years and above, with a history of use of PPI for at least one month, presenting to the medicine outpatient department or admitted to medicine wards were included after informed consent. Patients on dialysis, taking magnesium or diuretics, pregnant women, or having a BMI < 18 kg/m^2^ were excluded.

### Ethical Approval:

The study was approved by the ethical review board (Ref. No.: 1134; dated February 3, 2023).

Participants were assessed with a detailed medical history, covering PPI use duration and types, hypertension, and diabetes mellitus. Height, weight, and BMI were recorded. Blood samples were taken for serum magnesium assessment, centrifuged within two hours, and analyzed with a photometric and ion-selective electrode system. Hypomagnesemia was defined as serum magnesium below 1.7 mg/dl.^14^

### Statistical analysis:

Statistical Package for Social Sciences (SPSS) version 27 was used for data analysis. Descriptive statistics were used to summarize the characteristics of the participants. The chi-square test was applied to find the association between gender, outpatient-inpatient, presence/absence of hypertension and diabetes, duration of use of PPIs, and hypomagnesemia. Student t-test was employed to compare mean age and mean BMI in patients with and without hypomagnesemia, while ANOVA was used to compare serum magnesium across the types of PPI. A logistic regression model was used to further investigate the association between the variables after adjusting for confounders, and adjusted odds ratios and 95% confidence intervals were calculated. A p-value of less than 0.05 was regarded as statistically significant.

## RESULTS

Of the total 303 patients, 149 (49.2%) were male and 154 (50.8%) females, with a mean age of 43.7 ± 12.9 years (age ranges 19 to 72 years), and mean BMI of 25.5 ± 4.5 kg/m^2^. Most (71.6%) patients had used PPI for more than three months. Hypomagnesemia was seen in almost half of the patients in this study (n=156, 51.5%). The demographic and clinical parameters have been outlined in Table-I.

**Table-I:** Demographic parameters of the study population (n=303)

Variables	Mean ± SD / (n,%)
Age, Mean ± SD (years)	43.7 ± 12.9
BMI, Mean ± SD (kg/m^2^)	25.5 ± 4.5
*Gender (n,%)*	
Male	149 (49.2%)
Female	154 (50.8%)
*Patients’ Setting (n,%)*	
Outpatient	223 (73.6%)
Inpatient	80 (26.4%)
*Duration of PPI use (n,%)*	
Less than 3 months	86 (28.4%)
3 to 6 months	79 (26.1%)
More than 6 months	138 (45.5%)
*Type of PPI used (n,%)*	
Omeprazole	51 (13.5%)
Esomeprazole	154 (50.8%)
Dexlansoprazole	55 (18.2%)
Pantoprazole	53 (17.5%)
*Hypertension (n,%)*	
Yes	72 (23.8%)
No	231 (76.2%)
*Diabetes Mellitus (n,%)*	
Yes	73 (24.1%)
No	230 (75.9%)
*Hypomagnesemia (n,%)*	
Yes	156 (51.5%)
No	147 (48.5%)

SD: Standard Deviation, BMI: Body mass index, PPI: Proton pump inhibitor.

**Fig.1:**
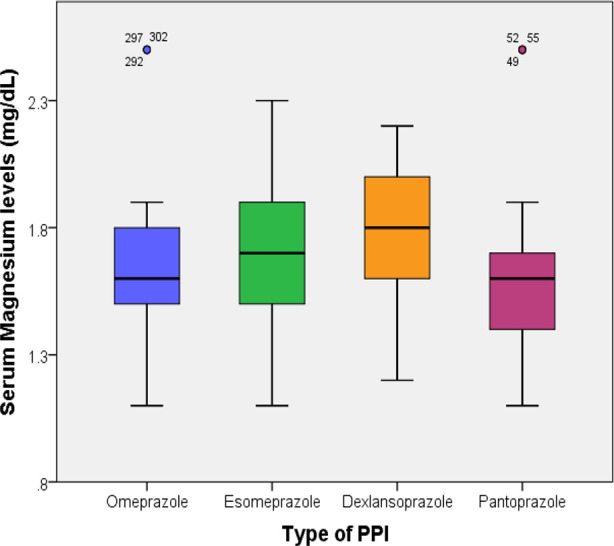
Relationship between type of PPI and serum magnesium levels (p=0.131).

Compared to patients without hypomagnesemia, those with hypomagnesemia had a significantly higher mean age (48.9 ± 12.3 vs 38.2 ± 11.4 years, p <0.001) and significantly higher mean BMI (26.2 ± 4.6 vs 24.7 ± 4.3 kg/m^2^, p=0.005). Hypomagnesemia was observed in a significantly higher proportion of patients (82.6%) who had used PPI for more than six months compared to those with PPI use for three to six months and less than three months (18.6% and 32.9%, respectively, p < 0.001). Similarly, a higher proportion of patients with diabetes mellitus had hypomagnesemia (78.1% vs 43%, p < 0.001). Gender, BMI category, patients’ setting, and hypertension did not have a significant association with hypomagnesemia. (Table-II). There was no significant difference in the serum magnesium levels among patients on different PPIs (p=0.131) (Fig.1).

**Table-II:** Association of demographic and clinical parameters with hypomagnesemia

Variables	Hypomagnesemia		*p-value
	No (n=147)	Yes (n=156)	
Age (years) (mean ± SD)	38.2 ± 11.4	48.9 ± 12.3	<0.001
BMI (kg/m^2^) (mean ± SD)	24.7 ± 4.3	26.2 ± 4.6	0.005
*BMI categories (kg/m^2^) (n,%)*			
Normal (<23)	60 (54.5%)	50 (45.5%)	0.113
Overweight and obese (≥23)	87 (45.1%)	106 (54.9%)
*Gender (n,%)*			
Male	80 (53.7%)	69 (46.3%)	0.076
Female	67 (43.5%)	87 (56.5%)
*Patient setting (n,%)*			
Outpatient	115 (51.6%)	108 (48.4%)	0.076
Inpatient	32 (40%)	48 (60%)
*Duration of PPI use (n,%)*			
Less than 3 months	70 (81.4%)	16 (18.6%)	<0.001
3 to 6 months	53 (67.1%)	26 (32.9%)	
More than 6 months	24 (17.4%)	114 (82.6%)
*Hypertension (n,%)*			
No	117 (50.6%)	114 (49.4%)	0.183
Yes	30 (41.7%)	42 (58.3%)
*Diabetes mellitus (n,%)*			
No	131 (57%)	99 (43%)	<0.001
Yes	16 (21.9%)	57 (78.1%)	

SD: Standard Deviation, BMI: Body mass index,

* p<0.05 was considered statistically significant.

Compared to patients with a history of PPI use for less than three months, those using PPIs for three to six months, and more than six months were 3.3 times (95% CI: 1.3 – 7.9, p < 0.001) and 58.1 times (95% CI: 22.2 – 152.5, p < 0.001) more likely to have hypomagnesemia, respectively. The likelihood of hypomagnesemia was also significant in patients with diabetes mellitus (AOR: 23.1, 95% CI: 6.4 – 82.6, p < 0.001) and hypertension (AOR: 3.2, 95% CI: 1.4 – 7.1, p < 0.001) when adjusted for other risk factors (Table-III).

**Table-III:** Results of multivariate logistic regression analysis of factors associated with hypomagnesemia

Variables		AOR	95% CI	p-value
*Gender*	Male*			
	Female	1.7	0.9 – 3.3	0.116
*Patients’ setting*	Outpatient*			
	In-patient	0.4	0.1 – 1.2	0.105
*Duration of PPI use*	Less than 3 months*			
	3 to 6 months	3.3	1.3 – 7.9	<0.001
	More than 6 months	58.1	22.2 – 152.5	<0.001
*Hypertension*	No*			
	Yes	3.2	1.4 – 7.1	<0.001
*Diabetes*	No*			
	Yes	23.1	6.4 – 82.6	<0.001
*BMI Categories*	Normal*			
	Overweight and obese	0.6	0.3 – 1.3	0.226

* Reference categories, AOR: Adjusted Odds Ratio, CI: Confidence Interval, BMI: Body Mass Index.

## DISCUSSION

Proton pump inhibitors are available over the counter in Pakistan and are often continued by patients beyond the prescribed duration. Some of the adverse effects of PPIs like hypomagnesemia may have fatal consequences. This study aimed at uncovering the extent of hypomagnesemia in patients using PPIs.

Overall, 51.5% of the patients in this study had hypomagnesemia. This is consistent with the findings of Alhosaini et al. who reported hypomagnesemia in 55.2% of patients using PPIs.^9^ Bahtiri et al. and Siddiqi et al. have reported a lower frequency of hypomagnesemia (2.3% and 3.6%, respectively).^8^,^11^ The discrepancy is due to variations in defining hypomagnesemia, study design, sample size, and PPI usage time frames. Older individuals were more likely to have hypomagnesemia with prolonged PPI use, similar to trends seen in studies from the Netherlands and Turkey.^15^,^16^

Patients with hypomagnesemia had a higher mean BMI, but no significant difference was found between normal and overweight/obese patients in both univariate and multivariate analyses. Conversely, Seah et al. in their retrospective study in Singapore reported a significant association between lower BMI and hypomagnesemia in PPI users.^17^ A smaller sample size in their study and differences in inclusion criteria could account for this difference.

The study found a strong link between low serum magnesium levels and prolonged use of PPIs. Patients using PPIs for extended periods were more likely to have hypomagnesemia. This is consistent with other research, such as Kim et al., who also found significant hypomagnesemia in long-term PPI users (p=0.042).^18^ This study also found that diabetic PPI users were more likely to have hypomagnesemia. This aligns with existing literature, which reports a 32% prevalence of hypomagnesemia in diabetic patients.^19^ Seah et al. also reported diabetes as being significantly associated with hypomagnesemia in PPI users.^17^

There was no significant association found between gender and hypomagnesemia in this study. This reflects the results of Shah DU et al. and Malik et al. in their studies carried out in India and Pakistan, respectively.^20^,^21^ Limited literature exists on the link between PPI types and hypomagnesemia. This study compared four PPIs and found no significant differences in serum magnesium levels. These results align with previous research showing similar hypomagnesemia across different PPIs.^17^ Hypomagnesemia is likely a class effect for all the different types of PPIs.

This is the first Pakistani study identifying predictors of hypomagnesemia in PPI users. Both inpatients and outpatients were included regardless of the PPI type. Logistic regression was used to control confounding factors, enhancing the validity of the results. This study fills a gap in literature and offers insights relevant to the local population.

### Limitations:

It includes a small sample size from a single hospital, which may limit applicability. The cross-sectional design establishes associations, not causation. Despite adjustments for confounders, unmeasured factors may still influence results. Relying on a single serum magnesium measurement may not accurately reflect long-term magnesium status.

## CONCLUSION

Nearly half of the patients on PPIs for at least one month had hypomagnesemia. Higher age, longer PPI use, and diabetes mellitus are key risk factors for hypomagnesemia. All PPIs predispose to hypomagnesemia, indicating a class effect. Physicians should be cautious with PPI prescriptions and should discourage self-medication with PPIs. Further studies with larger, more diverse populations and longitudinal designs are needed to better understand the relationship between PPI use and hypomagnesemia.

### Recommendations:

Several clinical implications arise from this study. First and foremost is the need for awareness about the consequences of prolonged use of a common, over-the-counter drug like PPIs. Secondly, the study advocates for the screening of all patients on long-term PPIs for hypomagnesemia. Lastly, there is a need for comparative studies on larger populations to validate the findings of this study.

### Authors Contribution:

**KNH** designed the study, did the data collection, and statistical analysis, and drafted and revised the manuscript.

**NH and AB** have collected data and drafted and reviewed the manuscript.

**FR** conceived the study, did statistical analysis, interpreted the results, and drafted and revised the manuscript.

All authors have approved the manuscript for publication and have agreed to be responsible for the integrity of the manuscript.
